# Genetic Therapy and Molecular Targeted Therapy in Oncology: Safety, Pharmacovigilance, and Perspectives for Research and Clinical Practice

**DOI:** 10.3390/ijms23063012

**Published:** 2022-03-10

**Authors:** Sabrina Orzetti, Federica Tommasi, Antonella Bertola, Giorgia Bortolin, Elisabetta Caccin, Sara Cecco, Emanuela Ferrarin, Elisa Giacomin, Paolo Baldo

**Affiliations:** 1 Hospital Pharmacy Unit of the “Centro di Riferimento Oncologico (CRO) di Aviano IRCCS”, Via F. Gallini, 33081 Aviano, Italy; sabrina.orzetti@cro.it (S.O.); federica.tommasi@cro.it (F.T.); antonella.bertola@cro.it (A.B.); giorgia.bortolin@cro.it (G.B.); elisabetta.caccin@cro.it (E.C.); ims@cro.it (S.C.); egiacomin@cro.it (E.G.); 2 Department of Hospital Pharmacy, Azienda Sanitaria Universitaria Friuli Centrale (ASUFC), 33100 Udine, Italy; 3 Scientific and Patients Library of the “Centro di Riferimento Oncologico (CRO) di Aviano IRCCS”, Via F. Gallini, 33081 Aviano, Italy; people@cro.it

**Keywords:** genetic therapy, targeted therapy, pharmacovigilance, cancer

## Abstract

The impressive advances in the knowledge of biomarkers and molecular targets has enabled significant progress in drug therapy for crucial diseases such as cancer. Specific areas of pharmacology have contributed to these therapeutic outcomes—mainly targeted therapy, immunomodulatory therapy, and gene therapy. This review focuses on the pharmacological profiles of these therapeutic classes and intends, on the one hand, to provide a systematic definition and, on the other, to highlight some aspects related to pharmacovigilance, namely the monitoring of safety and the identification of potential toxicities and adverse drug reactions. Although clinicians often consider pharmacovigilance a non-priority area, it highlights the risk/benefit ratio, an essential factor, especially for these advanced therapies, which represent the most innovative and promising horizon in oncology.

## 1. Introduction

In the last 20 years, pharmacology has attained incredible progress. The recent, unexpected, and fast implementation of a vaccination strategy against the SARS-CoV-2 virus in less than a year clearly confirms this. Evolution has also been remarkable in cancer treatment, improving 5-year overall survival (OS) from diagnosis by an average of more than 11% over a decade (for both male and female patients). For instance, Italian data shows, in the period 1990–1994, a 5-year survival rate for male patients of 39%, whereas, in 2005–2009, the same rate reached 54%; for female patients, in 1990–1994, the 5-year survival rate was 55% and, in 2005–2009, it increased to 63% [1].

Several areas of application in pharmacology have contributed to these therapeutic results—mainly targeted therapy and immunomodulatory therapy; other disciplines, such as gene therapy, pharmacogenomics, and vaccine therapy in oncology, are in constant development but need further confirmation by clinical research. In addition, the increased availability of oral preparations has made patient treatment possible not only in hospital oncology wards but also at home, thus promoting patient compliance. 

## 2. Study Selection

The selection of studies included in this review was performed by searching PubMed, Embase, the Cochrane Library, Clinicaltrials.gov, and websites of specific oncology medical associations: American Society of Clinical Oncology, European Society of Medical Oncology, and Associazione Italiana di Oncologia Medica. The studies selected to be included in this review used a multiplicity of terms to indicate the same pharmacological class; therefore, for the sake of clarity and consistency, preliminary definitions of the terms used in this review to group the pharmacological classes in the same clinical–therapeutic area are herein outlined.

## 3. Terms and Definitions Used

We used the definitions based on the official terms reported in the MeSH dictionary, provided by the National Library of Medicine [2]:

- Precision medicine (MeSH unique ID: D057285; year introduced: 2010), also often referred to as personalized medicine. Official definition: clinical, therapeutic, and diagnostic approaches to optimal disease management based on individual variations in a patient’s genetic profile.
- Genetic therapy (MeSH unique ID: D015316; year introduced: 2012), also often referred to as gene therapy in the medical literature. Official definition: techniques and strategies that include coding sequences and other conventional or radical means to transform or modify cells to treat or reverse disease conditions.
- Cell- and tissue-based therapy (MeSH unique ID; D064987, year introduced: 2014). Official definition: therapy that involves the transplantation of cells or tissues developed to restore the function of diseased or dysfunctional cells or tissues.
- Molecular targeted therapy (MeSH unique ID: D058990; year introduced: 2011), also often referred to as target therapy. Official definition: treatment with drugs that interact with or block the synthesis of specific cellular components characteristic of the individual’s disease to stop or interrupt the specific biochemical dysfunction involved in the progression of the disease.
- Antineoplastic agents (MeSH unique ID: D000970; year introduced: N/A). Official definition: substances that inhibit or prevent the proliferation of neoplasms.
- Immunotherapy (MeSH unique ID: D007167; year introduced: 1973). Official definition: manipulation of the host’s immune system in the treatment of disease. Includes both active and passive immunization as well as immunosuppressive therapy to prevent graft rejection.

## 4. Genetic Therapy for Cancer

**Figure 1:**
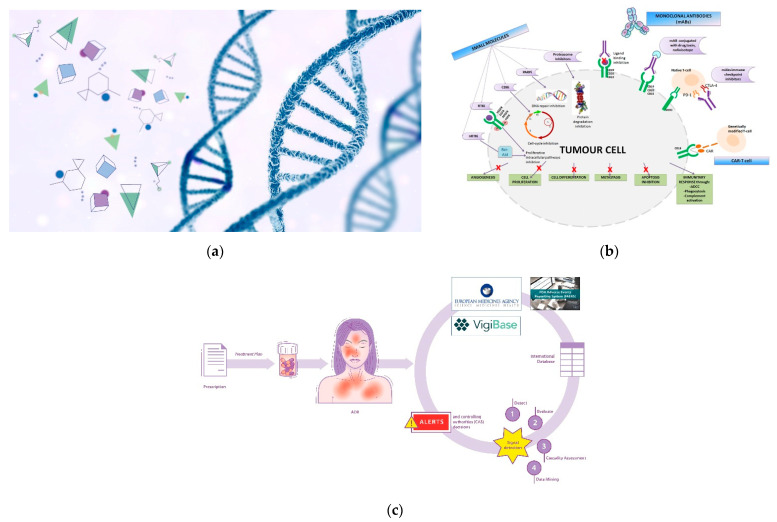
(**a**) Genetic therapy involves the interaction between pharmacological molecules and the genetic material of the cell; (**b**) Targeted therapy involve specific sites to interact with molecular targets in the cell. BCR-ABL: Breakpoint Cluster Region-Abelson gene; nRTKi: Non-Receptor Tyrosine Kinase inhibitors; RTKi: Receptor Tyrosine Kinase inhibitors; VEGFR: Vascular Endothelial Growth Factor Receptor; EGFR: Epidermal Growth Factor Receptor; PDGFR: Platelet-Derived Growth Factor Receptor; FGFR: Fibroblast Growth Factor Receptor; CDKi: Cyclin-Dependent Kinase inhibitors; PARPi: Poly Adenosine diphosphate-Ribose Polymerase inhibitors; MHCI: Major Histocompatibility Complex; PD-1: Programmed cell Death Protein 1; CTLA-4: Cytotoxic T-Lymphocyte Antigen 4; CAR: Chimeric Antigen Receptor; ADCC: Antibody-Dependent Cellular Cytotoxicity. (**c**) Pictorial rappresentation of the workflow of the International sistem of pharmacovigilance. Spontaneous reports of ADRs (adverse drug reactions) are collected from international databases (Vigibase, FAERS system and Eudravigilance) in order to generate alerts and implement post-marketing drug surveillance.

Genetic therapy is a promising and articulated research track in the oncology field. Although it is not currently commonly used in all hospitals/clinics, the scientific and technological concepts underlying it are highly refined and innovative (Figure 1a). Suffice it to say that one of the technologies (mRNA) that made vaccines against the SARS-CoV-2 virus -responsible for the current global pandemic- possible, also derived significantly from cancer research carried out in recent decades [3,4]. The pharmacotherapeutic classes concerning the field of oncology that can be included in the definition of gene therapy are oligonucleotides, oncolytic virus therapy, cell and tissue therapy, and specific vaccines for cancer [5,6]. However, it is not possible to categorically draw boundaries between the various definitions; for example, CAR-T therapy also acts on the immune system, and aptamers exert their therapeutic action as a function of their affinity with biocellular targets [7]. Similarly, immunotherapeutic agents exert their action by interacting with specific cellular target antigens. The utility in the classification of therapeutic agents is often functional, toward greater comprehensibility and schematic representation [8].

### 4.1. Oncolytic Viruses (OVs)

Viruses interact biologically with human cells in vivo, expressing selectivity for cancer cells and killing them. This is why they are then referred to as “oncolytic”. Although this type of approach is now included in the context of gene therapy against cancer, research in oncolytic viruses (OVs) has its origin in the early 1950s [9]. Although they belong to different families (Adenoviridae, Herpesviridae, Paramyxoviridae, Parvoviridae, Picornaviridae, Poxviridae, Reoviridae, Rhabdoviridae) [10], there are essentially three viral agents currently registered for therapeutic application, the first of which was Rigvir^®^ in 2004 [11], while many agents are under investigation for use in diagnostic and therapeutic techniques in different types of cancer [12]. OVs are engineered to infect cancer cells, replicate, and cause cell lysis while sparing healthy cells. In addition to this mechanism of action, OVs contribute to the global response of the organism by expressing substances and antigens in the tumor microenvironment (MEV). They also contribute to an organic/biological reactivity that can be exploited for more accurate diagnosis by aiding in the use of imaging technologies (e.g., fluorescence, luminescence) [13,14,15].

### 4.2. Cell- and Tissue-Based Therapy for Cancer

Although this product category can be considered borderline in terms of immunotherapy (a sort of cell immunotherapy), it best represents innovation in the field of biotechnology and advanced therapy. Chimeric antigen receptor (CAR-T) cells and T cell redirecting bispecific T cell engager (BiTE) are approved for use in several forms of hematologic malignancies [16]. The main concept underlying the mechanism of action of this class of drugs is the redirection of T cell reactivity against specific tumor antigens. CAR-T cells are genetically engineered T cells with a chimeric antigen receptor [17,18]. The CAR is composed of an extracellular single-chain variable fragment (scFv), a domain that recognizes tumor-specific antigens and intracellular signaling targets. BiTEs are recombinant proteins consisting of two scFv fragments of separate antibodies, one to target a tumor-specific antigen and one to intercept and recruit active T cells. Recruited T cells are then redirected to kill cancer cells.

Select clinical trials investigating the safety of genetic therapy for cancer are reported in Appendix A (Table A1).

### 4.3. Cancer-Specific Vaccines

Although the therapeutic potential of vaccines in the treatment of various forms of cancer has yet to be attained, the technologies used for the rapid development of vaccines against the SARS-CoV-2 virus, particularly viral-vector and DNA/RNA-based technologies, derive from decades of scientific and laboratory research in the fight against cancer [19]. The success of prophylactic strategies against pathogens such as polio and smallpox viruses, or viral-driven cancers such as hepatitis B virus (HBV), which causes hepatocarcinoma, and human papilloma virus (HPV), which causes cervical cancer [20,21], suggests potential new perspectives for the development of “preventive” (or prophylactic) anticancer vaccines. Still, so far, research has not yielded satisfactory results for other forms of cancer.

In parallel, the class of anticancer vaccines, defined as therapeutic, includes agents that belong to several categories: cell-, peptide-, DNA- or RNA-, viral-vector, or bacterial-vector based vaccines [22,23]. To quickly understand the potential benefits expected of gene therapy through this category of agents, we must bear in mind that the goal of any therapeutic cancer vaccine is to increase and reactivate the body’s latent immune response, specifically that of non-active T cells in the tumor microenvironment, by stimulating dendritic cells (DCs), thus conferring the T cells with the property of being tumor-specific antigens (TAAs). Moreover, efficient delivery of vaccines is required through nanocarriers or specific adjuvant molecules or ligands that favor an effective interaction with the tumor microenvironment and, therefore, the release of therapeutic or cytotoxic agents to specific cellular targets.

Select clinical trials investigating the safety of vaccines for cancer are reported in Appendix A (Table A2). 

### 4.4. Combination Therapies and Therapeutic Oligonucleotides

Several studies are currently evaluating combination therapy strategies involving various agents, immunomodulatory therapy, bi-specific T cell engagement, cell-tissue therapy with CAR-T, and the use of specific vaccines targeted to various forms of cancer. Recent reviews by Shi et al. [24] and Chaurasiya et al. [25] have presented comprehensive summaries of ongoing studies on oligonucleotides/aptamers. Since 1990, technologies involving antisense oligonucleotides (ASOs), aptamers, microRNA (miRNAs), small interfering RNAs (siRNAs), and catalytic DNA with enzymatic properties (DNAzymes) have been investigated to uncover new therapeutic possibilities and overcome some of the limitations in the curative potential of monoclonal antibodies (mAbs) and targeted therapy. These are considered promising approaches to the treatment of resistant types of cancer [26]. Therapeutic oligonucleotides/aptamers interact with target cells, causing RNA alterations/modifications by several different mechanisms (mRNA degradation, pre-mRNA splicing, or mRNA translation) [27]. Besides being a potential strategy for cancer therapy, the use of oligonucleotides holds promise for treating also many forms of illness due to genetic aberrations (for example, neurological and ocular diseases). They also deserve to be used clinically in diagnostic procedures, such as liquid biopsy [28,29,30]. Safety may be a major concern for this type of molecule, along with a lack of efficacy, potentially due to difficulty in delivering the active components to the site of action.

## 5. Pharmacovigilance and Adverse Drug Reactions (ADRs) of Genetic Therapy for Cancer

Since the fundamental principle behind these advanced and innovative therapies is to hit molecular targets in tissues or organs affected by cancer, toxicity is best understood with the concept of “off-target, off-organ” toxicity. This concept well explains the possibility of originating immunological reactions and cell lysis at a systemic level, and triggering the release of a cascade of substances, for example, cytokines [31]. Early identification of the adverse events and awareness about their management is critical for the implementation of the use of these innovative therapies in clinical practice.

A relevant conditioning factor is undoubtedly the very high cost of products such as CAR-T, which involve cellular engineering and adequate clinical setting conditions; since research is very expensive, the possibilities for study protocols are limited, and consequently, clinical safety data are often scarce or inconsistent [32,33,34].

### 5.1. Oncolytic Viruses

Oncolytic virotherapy is associated mainly with flu-like symptoms and local reactions at the injection site. Flu-like symptoms include fever, chills, nausea, fatigue, myalgia, and gastrointestinal symptoms such as diarrhea. Symptomatology appears to be dose-related and ADRs can be mitigated with the administration of acetaminophen or steroids [35]. Dermatological manifestations have also been observed, including rash, erythema, and edema. Other reported relevant responses to toxicity are hematological abnormalities (thrombocytopenia, leukopenia, neutropenia), cardiovascular disorders (arrhythmia, hypotension), and central nervous system (CNS) disorders (seizures, speech disorders, disorientation). 

### 5.2. Cell- and Tissue-Based Therapy

The most frequent and severe toxicities associated with CAR-T cell therapy are: cytokine release syndrome (CRS), neurotoxicity, B cell aplasia and hypogammaglobinemia. Among these events, the most limiting remain CRS syndrome and the immune effector cell-associated neurotoxicity syndrome (ICANS).

In summary, the incidence of CRS is reported to range from 25 to 80%, while for ICANS, from 50 to 70% [36,37]. These potentially life-threatening responses are associated, respectively, with targeted cell lysis and related electrolyte imbalance. Myeloid cells exposed to CAR-T treatment rapidly release inflammatory mediators, proteins, and cytokines such as GM-CSF, C-reactive protein, IL-6, and IL-1b [38,39]. A series of organ-specific adverse reactions can be exacerbated by CRS, for example, hyperthermia, myalgia, abdominal pain, dyspnea, hypotension, arrhythmia, erythema, pruritus, renal failure, and granulocytopenia. Early identification and management can mitigate the severity of adverse effects and reduce mortality. Suggestive symptoms for early identification include fever, hypotension, hypoxia, rash, headache, respiratory and breath shortness, coagulopaty, and organ failure. 

In 2019 and late 2021, respectively, multidisciplinary expert panels of the American Society for Transplantation and Cellular Therapy (ASTCT) and the American Society of Clinical Oncology (ASCO) released new guidelines, proposing a series of actions for the management of the main adverse events caused by the use of CAR-T cell [40,41]. 

Key recommendations include early management of grade 1 or short term toxicities related to CAR-T cell treatment, management of patients with severe or prolonged toxicities, by administrating tociluzumab with or without corticosteroids [42]; treatment with corticosteroids and/or best supportive care until improvement of patient’s condition or resolution of adverse reactions. Tocilizumab acts as an antagonist of IL-6 receptor and is able to block the cascade of IL-6 in CRS, preventing IL-6 from binding to its receptors through competitive inhibition, neutralizing the activity of IL-6 signaling [43]. The prophylactic use of tocilizumab administered 1 h prior to the infusion of CAR-T cell, has been investigated, showing promising results, reducing CRS incidence and severity [44]. Car-T cell therapies are associated also with cardiovascular toxicities. They include hypotension, tachycardia, atrial fibrillation, ventricular dysfunction, and cardiac failure. Patients with pre-existing risk factors to CAR-T treatment need to be appropriately monitored [45]. Biomarkers (i.e., troponins, natriuretic peptides, nitric oxid metabolites, and microRNAs) also appear to be important for the detetection of patients at increased risk of CAR-T cardiotoxicity, both in adult and pediatric patients [46,47].

Concerning the use of other classes in the field of gene therapy, namely BiTES or bi-specific antibodies (Bi-Abs), several studies and pre-clinical research are currently ongoing [48], and toxicity similar to that of non-bi-specific antibodies and immunotherapy can be expected [49]. 

### 5.3. Vaccines

The main issues concerning vaccines do not seem related to their toxicity and safety but mostly to understanding which specific forms of cancer and in which therapeutic combinations they prove the best efficacy. Our experience with vaccines to prevent SARS-CoV-2 infection at an international level shows that they have a highly favorable safety profile, with some rare fatal events, considering the vast number of people vaccinated worldwide in the post-marketing setting. It must be highlighted that the biopharmaceutical technology that led, in a very short time, to the development of COVID vaccines (i.e., mRNA vaccines) comes from cancer immunotherapy research [3,4,5,6,7,8,9,10,11,12,13,14,15,16,17,18,19,20,21,22,23,24,25,26,27,28,29,30,31,32,33,34,35,36,37,38,39,40,41,42,43,44,45,46,47,48,49,50]. Commonly detected toxic responses following cancer vaccine administration include myalgia, cough, chills, fever, pain at the injection site, asthenia, flu-like syndrome, and respiratory abnormalities [20,51,52,53]. 

## 6. Targeted Therapy for Cancer

Targeted therapy has raised new questions regarding the personalization of anticancer treatment, the evaluation of drug efficacy and toxicity, and the economics of treatment. Identifying specific molecular targets has led to the development of targeted therapy in cancer treatment [54,55] (Figure 1b). These drugs have allowed us to expand the concept of individualized cancer treatment, since they act only in patients with that specific genetic profile and mutation. Alterations in the genetic profiles that cause mutations in proteins or receptors involved in cell survival and proliferation underlie tumor development. These specific genetic alterations distinguish normal cells from diseased cells, and subsequently, treatment is targeted primarily to cancerous cells [56]. The recent improvements in this field have led to new clinical trial designs such as basket trials [54]. These studies evaluate targeted therapy for multiple diseases that share common molecular alterations or risk factors. Basket trials assess a single investigational drug or drug combination in different oncological populations that differ in several factors: disease stage, histology, number of prior therapies, genetic or other biomarkers, or demographic characteristics [57,58].

In clinical practice, targeted therapy is used in breast, colorectal, lung, and pancreatic cancer as well as in lymphoma, leukemia, and multiple myeloma [55].

Targeted therapy is aimed at growth factors, signaling molecules, cell cycle proteins, apoptosis modulators, and molecules promoting angiogenesis [59]. The two main types of targeted therapy are monoclonal antibodies and small molecule inhibitors. 

Select clinical trials that investigated their safety are reported in Appendix A (Table A3).

### 6.1. Monoclonal Antibodies

In 1986, the Food and Drug Administration approved the first monoclonal antibody, muromonab-CD3, which prevented organ rejection after transplantation by blocking T cell action [55]. Within a short period, monoclonal antibodies entered the mainstream of anticancer therapy. The first approved mAbs were directed against targets expressed in solid tumors selected in cell culture, such as EGFR antagonists and HER2 [60,61]. 

Immunoglobulins exert anticancer action through various mechanisms: by binding ligands or receptors, thereby interrupting the process of oncogenesis; by transporting lethal molecules such as radioisotopes or toxins to the target cell [55] and triggering the immune response to attack cancer cells and fight the disease (immunotherapy). Specifically, immunotherapy provides a therapeutic benefit in fighting some types of cancer by activating the immune system; it acts by blocking cytotoxic T lymphocyte antigen 4 (CTLA-4), programmed cell death receptor (PD-1), and chimeric antigen receptor T cells (CAR-T) in favor of the immune response and thus the elimination of cancer cells [62]. 

In recent decades, much progress has been made in understanding how cancer evades the immune response by offering new ways to stop the immune evasion of cancer in favor of eliminating cancer cells. Table 1 summarizes individual targets of anticancer monoclonal antibodies together with the therapeutic indications. 

### 6.2. Small Molecule Inhibitors

Small molecule inhibitors are low-molecular-weight compounds (<900 Da) that can enter cells and block specific target proteins. Most small molecule inhibitors inactivate kinases by interrupting the signaling pathway that is dysregulated during carcinogenesis; they can also target the proteasome, cyclin-dependent kinases (CDKs), and poly (ADP-ribose) polymerases (PARPs) [64]. The main targets of small molecule inhibitors are tyrosine kinase receptors: epidermal growth factor receptor (EGFR), vascular endothelial growth factor (VEGF), and human epidermal growth factor 2 (HER2/neu). These pathways can be inhibited on different levels: by binding and neutralizing the ligand, occupying the receptor binding site, blocking signal transduction in the cancer cell, or interfering with molecules involved in downregulation [65]. Table 2 shows the small molecules that are currently approved in oncology clinical practice by the European Medicines Agency. 

## 7. Pharmacovigilance and Adverse Drug Reactions of Targeted Therapy for Cancer

Several studies have shown that targeted therapy could improve the OS, progression-free survival (PFS), and response rate (RR) of cancer patients [66]. This is partly due to the lower risk of developing lethal ADRs compared to standard chemotherapy. Studies have shown that approximately 32% of patients treated with targeted therapy had to discontinue due to severe ADRs, which led to poor prognoses, such as cancer recurrence or progression [67].

Although the rate of lethal ADRs is lower, targeted drugs are employed for prolonged periods or indefinitely, causing a high incidence (80%) of some ADRs during treatment [68]. ADRs from targeted therapy have the following main characteristics: (1) they occur in multiple organ systems; (2) different drugs cause different ADRs in terms of type, frequency, and severity; (3) most ADRs occur during the initial administration and remain stable throughout treatment; (4) most encountered ADRs are mild, therefore, easily manageable; and (5) some can cause discontinuation of therapy or other interventions. Therefore, it is important to monitor and manage ADRs during drug treatment to improve cancer patients’ adherence to therapy and prognosis [69].

According to recent studies, ADRs occur most frequently in the skin and mucous membranes (86.4%). Adverse reactions such as gastrointestinal symptoms, hypertension, coagulation disorders, and cardiotoxicity are also common. In order to improve treatment response, it is important to identify not only the known ADRs, but also, and especially, the events that are difficult to correlate [70].

## 8. Innovative Treatments: Advantages and Limitations

The guidelines recommend that drug treatments be chosen based on efficacy, tolerability, and other factors, and should include optimizing adherence and monitoring adverse side effects. Medical decision-making must also consider setting stage-specific treatment goals and improving the patient’s quality of life.

### 8.1. Genetic Therapy

Genetic therapies include highly targeted and individualized treatments such as CAR-T, obtained by cell engineering techniques, and advanced delivery techniques (such as viral vector delivery). They represent an essential breakthrough for clinical areas such as oncology, offering new care opportunities.

Gene therapy allows implementation of the “agnostic” therapeutic approach, i.e., the use of drugs that do not depend on the cellular isotype and the organic site of the tumor, but directly target cellular alterations/aberrations. Furthermore, each subclass of genetic therapy has inherent limitations. For example, oncolytic viruses can affect only viral antigens, limiting the effectiveness of OV therapy; the reaction and the immune response have significant individual variability, and a relevant problem is the rapid viral elimination of OVs from the organism [35,36,37,38,39,40,41,42,43,44,45,46,47,48,49,50,51,52,53,54,55,56,57,58,59,60,61,62,63,64,65,66,67,68,69,70,71]. In addition, CAR-T therapy requires a very complex lymphodepletion process, implemented with chemotherapy before infusion; the consequent risk of serious adverse events such as cytokine release syndrome calls for immediate management and advanced clinical skills [72]. Therefore, clinical trials to investigate these treatment strategies must be designed and planned for both the follow-up of patients and the available technologies, which require advanced clinical and hospital settings and vast investments of economic resources [73,74].

Because of these limitations, each advanced treatment option, as a single strategy, still needs to be optimized with further research and clinical evaluation [75].

### 8.2. Targeted Therapy

The main goal of cancer therapy is to act on target cells precisely without inflicting collateral damage on normal cells. To destroy rapidly dividing malignant cells, the mainstay of treatment has been the administration of small cytotoxic molecules with or without radiation therapy.

Targeted therapies have expanded the concept of tailored cancer treatment, because some of these drugs can be effective in patients whose tumors have a specific molecular target but may not be effective in the absence of that target. This distinction may be influenced by the ethnicity and sex of the patient and the histology of the tumor. In addition, targeted therapies require novel approaches to determine optimal dosing, assess patient adherence to therapy, and evaluate treatment efficacy [55].

In addition to extending patient survival, targeted therapies provide treatment options for some patients who might not otherwise be candidates for standard cancer therapy. For example, non-small-cell lung cancer and non-Hodgkin’s lymphoma primarily affect older patients, many of whom have medical comorbidities that limit the use of standard chemotherapy.

The protein structure of monoclonal antibodies is denatured in the gastrointestinal tract, so they should only be administered intravenously. They also have a short in vivo half-life after intravenous administration, requiring frequent or continuous infusions; they do not undergo hepatic metabolism. Therefore, they are not subject to significant drug interactions. The high cost of this therapy can be a major issue in the economic management of health care [61].

Small-molecule inhibitors differ from monoclonal antibodies. They are mainly administered orally rather than intravenously; compared to monoclonal antibodies, produced through an expensive bioengineering process, small molecules are chemically produced at a lower cost. This therapy, however, has limitations, as the molecules have a lower target specificity than monoclonal antibodies and are potentially subject to drug–drug interactions. Unlike monoclonal antibodies, most small-molecule inhibitors are metabolized by cytochrome P450 enzyme. Among the drugs with which they can interact are macrolide antibiotics, azole antifungals, some anticonvulsants, protease inhibitors, warfarin, and St. John’s wort.

Most small-molecule inhibitors have a half-life of a few hours and require daily dosing [55].

## 9. Pharmacovigilance Issues and Final Considerations

Toxicity on the one hand and the development of drug resistance on the other are the most significant issues affecting the efficacy of cancer therapies and, therefore, the desired therapeutic outcome. Although diverse, the toxicities of traditional cytotoxic chemotherapy and new targeted or genetic therapies are essentially due to the narrow therapeutic window, low selectivity between cancerous and healthy cells, and the need to use dosages close to the maximum tolerable. Similar to the concept of tumor and intratumor heterogeneity—one of the major problems in understanding and fighting cancer—the development of resistance to therapy is also due to multifactorial elements [76]. Numerous causes can nullify the therapeutic effect: host factors, for example, genetic variants and individual response to drugs and tumor factors, such as altered influx and outflow of drugs through cellular transporters, genetic modifications in molecular targets, increased DNA repair mechanisms, and changes in the tumor microenvironment [77]. It is worth mentioning that in the 1950s, similar observations, combined with Law and Skipper’s “mathematical” reasoning, formed the motivation to give rise to anticancer combination chemotherapies to circumvent resistance. [78]. Multifactorial resistance mechanisms cause changes in the pathways that regulate apoptosis, autophagy, and ultimately lead to cancer cell death [79,80]. Many studies currently focus on understanding specific mechanisms in specific cancers, such as breast, lung, and gastrointestinal cancers [81,82,83]. Further research must be warranted to ensure the efficacy of the most promising and innovative treatments, circumventing the phenomenon of resistance and favoring the path to the ultimate defeat of cancer.

In parallel with the immense development of pharmacology and immunotherapy, awareness of other important aspects that impact patient care and toxicity management has also increased significantly. In Europe, the implementation of renewed Pharmacovigilance legislation (Directive 2010/84/EU) has not only changed and specified the definition of “adverse reaction” (AR), but has also helped to implement electronic registers and the international network on which the recording of suspected ARs caused by drugs is based. With the renewed definition of AR, which is considered “a response to a medicinal product which is noxious and unintended”, the regulatory body intends to include any unwanted event resulting from the use of a drug, such as administration errors, or deriving from off-label use or misuse and abuse [84]. The new law (the workflow in Europe is represented in Figure 1c has also implemented the pharmacovigilance activity and risk management system in clinical settings, defining the system as “a set of pharmacovigilance activities and interventions designed to identify, characterize, prevent or minimize risks relating to a medicinal product, including the assessment of the effectiveness of those activities and interventions”. Nevertheless, so-called underreporting in Pharmacovigilance remains a relevant phenomenon, mainly due to the refusal or failure to report adverse events by health personnel. This is particularly evident in critical areas of medicine such as oncology, pediatrics, and emergency medicine [85].

## 10. Conclusions

Gene therapy and targeted molecular therapy can provide clinical results and outcomes in the oncology field that were unthinkable until a few decades ago. Classical chemotherapy, which unfortunately has never failed to cause a sort of systemic intoxication of the organism, is not wholly replaceable today in the context of effective and consolidated therapeutic protocols. Some effects are more specific than those of systemic chemotherapy, depending on the molecular targets involved (e.g., neurotoxicity and skin toxicity). Others are non-specific and may result from individual iatrogenic immunological responses, which, unfortunately, are often non-predictable. On the one hand, combined therapies and pharmacological synergies that can be exploited today enhance therapeutic efficacy; on the other hand, they seem to be able to reduce the resulting toxicity. It is essential that future research takes into consideration both the investment and the training of personnel who carry out clinical trials, and who have to manage increasingly sophisticated substances and drugs. Furthermore, regulatory bodies and health policymakers should be knowledgeable on how to appropriately allocate the volume of available resources, prioritize needs, and concurrently be able to make the most effective treatments available to the widest possible segment of the population.

## Figures and Tables

**Table 1:** Therapeutic monoclonal antibodies for cancer therapy currently marketed with regulatory approval from the European Medicines Agency (as of April 2020) [63].

mAbs	Target	Cancer Type
Mouse (-omab)		
Blinatumomab	CD19/Cd3 epsilon	Philadelphia chromosome-negative relapsed or refractory B cell precursor acute lymphoblastic leukemia
Ibritumomab tiuxetan	CD20	Non-Hodgkin lymphoma (non-HL)
Human-mouse chimeric (-ximab)		
Brentuximab vedotin	CD30	Hodgkin lymphoma (HL) after failure of stem cell transplantation or CT, Systemic anaplastic large cell lymphoma (sALCL) after failure of CT, post-auto-hematopoietic stem cell transplantation (HSCT) consolidation treatment for Hodgkin lymphoma (HL)
Cetuximab	^1^ EGFR	Colorectal, head, and neck cancers
Dinutuximab	^2^ GD2	Pediatric patients with high-risk Neuroblastoma
Rituximab	CD20	Non-Hodgkin lymphoma (Non-HL), chronic lymphocyticleukemia (CLL), rheumatoid arthritis, Wegener’s granulomatosis
Humanized (-zumab)		
Atezolizumab	^3^ PD-L1	Non-small cell lung cancer (NSCLC)
		Metastatic urothelial carcinoma (MUC)
Bevacizumab	^4^ VEGF-A	Metastatic colorectal cancer, non-squamous NSCLC, metastatic breast cancer, glioblastoma
Elotuzumab	^5^ SLAMF7	Multiple myeloma (MM)
Gemtuzumab ozogamicin	CD33	Acute myeloid leukemia (AML)
Inotuzumab ozogamicin	CD22	Acute Lymphoblastic Leukemia (ALL)
Mogamulizumab-kpkc	^6^ CCR4	Mycosis fungoides, Sézary syndrome
Obinutuzumab	CD20	In combination with chlorambucil for previously untreated chronic lymphocytic leukemia (CLL)
Pembrolizumab	^7^ PD-1	Unresectable or metastatic melanoma, refractory metastatic NSCLC tumors that express PD-L1
Pertuzumab	^8^ HER2	Combination with trastuzumab and docetaxel for HER2-positive metastatic breast cancer
Polatuzumab	CD79b	Diffuse large B cell lymphoma
Trastuzumab	HER2	Breast cancer overexpressing HER2, metastatic gastric or gastroesophageal (GE) junction adenocarcinoma overexpressing HER2
Ado-trastuzumab	HER2	HER2-positive breast cancer in patients who previously received trastuzumab or a taxan
Fully human (-umab)		
Avelumab	PD-L1	Merkel-cell carcinoma (MCC), ulcerative colitis (UC), renal cell carcinoma (RCC)
Cemiplimab-rwlc	PD-1	Cutaneous squamous cell carcinoma (CSCC)
Daratumumab	CD38	Multiple myeloma (MM)
Denosumab	RANKL	Giant cell tumor of bone, bone loss
Durvalumab	PD-L1	Non-small cell lung cancer (NSCLC)
Ipilimumab	^9^ CTLA-4	Metastatic melanoma
Nivolumab	PD-1	Unresectable or metastatic melanoma and disease progression following ipilimumab and, if BRAF V600 positive, a BRAF inhibitor, non-small cell lung cancer (NSCLC)
Olaratumab	^10^ PDGFR-alfa	Soft tissue sarcoma
Panitumumab	EGFR	Metastatic colorectal cancer
Ramucirumab	^11^ VEGFR2	Gastric or GE junction adenocarcinoma, metastatic NSCLC with docetaxel after platinum therapy, hepatocellular carcinoma (HCC), with folfiri for metastatic colorectal cancer

^1^ EGFR, epidermal growth factor receptor; GD2, ^2^ GD2 disialoganglioside; ^3^ PD-L1, programmed death-ligand 1; ^4^ VEGF-A, vascular endothelial growth factor A; ^5^ SLAMF7, signaling lymphocytic activation molecule family; ^6^ CCR4, C-C motif chemokine receptor 4; ^7^ PD-1, programmed death 1; ^8^ HER2, human epidermal growth factor receptor 2; ^9^ CTLA-4, cytotoxic T-lymphocyte antigen 4; ^10^ PDGFR-alfa, platelet-derived growth factor receptor alpha; ^11^ VEGFR2, vascular endothelial growth factor receptor 2.

**Table 2:** List of European Medicines Agency approved small molecule inhibitors used in clinics [63].

Small Molecule	Target	Cancer Type
Signal transduction inhibitors		
*Non-receptor tyrosine kinase inhibitors*		
Bosutinib	^1^ BCR-ABL	Chronic myeloid leukemia (CML)
Crizotinib	^2^ ALK kinase	Non-small cell lung cancer (NSCLC)
Dasatinib	BCR-ABL	Chronic myeloid leukemia (CML)
Imatinib	^3^ PDGFR, ABL kinase	Chronic lymphocyte leukemia (CLL)
Ibrutinib	^4^ BTK inhibitor	Chronic myelogenous leukemia (CML)
		Gastrointestinal stromal tumors (GIST)
		Mantle cell lymphoma (MCL)
Nilotinib	BCR-ABL	Chronic myeloid leukemia (CML)
Ponatinib	BCR-ABL	Chronic myeloid leukemia (CML)
*Receptor tyrosine kinase inhibitors*		
Afatinib	^5^ EGFR	Non-small cell lung cancer (NSCLC)
Erlotinib	EGFR	Non-small cell lung cancer (NSCLC)
Gefitinib	EGFR	Non-small cell lung cancer (NSCLC)
Gilteritinib	ALK kinase, AXL	Acute myeloid leukemia (AML)
Lapatinib	EGFR/^6^ ERBB2	ERBB2-positive breast cancer
Lenvatinib	^7^ VEGFR	Metastatic thyroid cancer
		Advanced renal cell carcinoma (aRCC)
Midostaurin	^8^ PKCalpha, ^9^ VEGFR2, PDGFR	Acute myeloid leukemia (AML)
Nintedanib	PDGFR, VEGFR, ^10^ FGFR	Non-small cell lung cancer (NSCLC)
Osimertinib	EGFR	Non-small cell lung cancer (NSCLC)
Sorafenib	VEGFR, ^11^ RAF, PDGFR	Advanced renal cell carcinoma (aRCC)
Sunitinib	VEGFR, PDGFR, ^12^ SCF	Hepatocellular carcinoma (HCC)
		Gastrointestinal stromal tumor (GIST)
		Advanced renal cell carcinoma (RCC)
Pazopanib	VEGFR, PDGFR, FGFR, SCF, ^13^ Itk, ^14^ Lck	Advanced pancreatic neuroendocrine tumors (pNET)
Regorafenib	VEGFR, PDGFR, FGFR	Advanced soft tissue sarcoma (STT)
		Advanced renal cell carcinoma (RCC)
		Metastatic colorectal cancer (CRC)
Vandetanib	VEGFR, EGFR, ^15^ RET	Advanced gastrointestinal stromal tumors
		Hepatocellular carcinoma (HCC)
		Metastatic medullary thyroid cancer (MTC)
Cabozantinib	VEGFR, RET, ^16^MET	Advanced renal cell carcinoma (aRCC)
		Hepatocellular carcinoma (HCC)
		Medullary thyroid cancer (MTC)
Proteasoma inhibitors		
Carfilzomib	Proteasome	Multiple Myeloma (MM)
Bortezomib	Proteasome	Multiple Myeloma (MM)
Ixazomib	Proteasome	Multiple Myeloma (MM)
Cyclin-dependent kinase (CDK) inhibitors		
Ribociclib	^17^ CDK4, ^18^ CDK6	Metastatic breast cancer
Palbociclib	CDK4, CDK6	Metastatic breast cancer
Abemaciclib	CDK4, CDK6	Metastatic breast cancer
Poly ADP-ribose polymerase (PARP) inhibitors		
Rucaparib	^19^ PARP	BRCA-positive ovarian cancer
Olaparib	PARP	gBRCA-mutated advanced ovarian cancer
Niraparib	PARP	Epithelial ovarian, fallopian tube, or primary peritoneal cancer

^1^ BCR-ABL, breakpoint cluster region-Abelson gene; ^2^ ALK, anaplastic lymphoma kinase; ^3^ PDGFR, platelet-derived growth factor receptor; ^4^ BTK, Bruton’s tyrosine kinase; ^5^ EGFR, epidermal growth factor receptor; ^6^ ERBB2, Erb-B2 receptor tyrosine kinase 2; ^7^ VEGFR, vascular endothelial growth factor receptor; ^8^ PKC alpha, protein kinase C alpha; ^9^ VEGFR2, vascular endothelial growth factor receptor 2; ^10^ FGFR, fibroblast growth factor receptor; ^11^ RAF, rapidly accelerated fibrosarcoma; ^12^ SCF, stem cell factor; ^13^ Itk, interleukin-2-inducible T-cell; ^14^ Lck, lymphocyte-specific protein tyrosine kinase; ^15^ RET, rearranged during transfection; ^16^ MET, mesenchymal epithelial transition factor; ^17^ CDK4, cyclin-dependent kinase 4; ^18^ CDK6, cyclin-dependent kinase 6; ^19^ PARP, poly adenosine diphosphate-ribose polymerase.

## Data Availability

Not applicable.

## Appendix A

**Table A1:** Select clinical trials investigating the safety of genetic therapy—19 September 2021 [74].

NCT Number	Title	Status	Phase	Conditions	Interventions	Outcome
NCT03125577	Combination CAR-T Cell Therapy Targeting Hematological Malignancies	Recruiting	1/2	•B-cell Malignancies	•Biological combinations: 4SCAR19 and 4SCAR22 4SCAR19 and 4SCAR38 4SCAR19 and 4SCAR20 4SCAR19 and 4SCAR123 4SCAR19 and 4SCAR70 4SCAR19 and 4SCAR30	•Safety of fourth-generation anti CD19 and CD20/CD22/CD30/CD38/CD70/CD123 CAR-T cells in patients with relapsed B cell malignancies using CTCAE 4 standard to evaluate the level of adverse events standard to evaluate the level of adverse events •Anti-tumor activity of fourth-generation anti CD19 and CD20/CD22/CD30/CD38/CD70/CD123 CAR-T cells in patients with relapsed or refractory B cell malignancies
NCT03849651	TCRαβ-depleted Progenitor Cell Graft With Additional Memory T-cell DLI, Plus Selected Use of Blinatumomab, in Naive T-cell Depleted Haploidentical Donor Hematopoietc Cell Transplantation for Hematologic Malignancies	Recruiting	2	•Acute lymphoblastic leukemia (ALL) •Acute myeloid leukemia (AML) •Myelodysplastic syndromes (MDS) •NK-cell leukemia •Hodgkin lymphoma •non-Hodgkin lymphoma (NHL) •Juvenile myelomonocytic leukemia (JMML) •Chronic myeloid leukemia (CML)	•Drug: Cyclophosphamide •Biological: Fludarabine •Drug: Thiotepa •Drug: Melphalan •Biological: G-csf •Drug: Mesna •Device: CliniMACS •Biological: ATG (rabbit) •Drug: Blinatumomab •Biological: TCRα/β+ •Biological: CD19+ •Biological: CD45RA-depleted DLI	•Maximum effective dose for prophylactic CD45RA-depleted DLI •One-year event-free survival (EFS) after completion of the protocol •The number of patients experiencing Blinatumomab permanent discontinuation due to toxicity •The estimate of cumulative incidence of relapse •The cumulative incidence of acute and chronic Graft-versus-host disease (GVHD) •The cumulative incidence of transplant-related mortality
NCT03765177	CLIC-1901 for the Treatment of Patients With Relapsed/Refractory CD19 Positive Hematologic Malignancies Title Acronym: CLIC-01	Recruiting	1/2	•Acute lymphoblastic leukemia •Non-Hodgkin’s lymphoma	•Biological: CLIC-1901	•Proportion of participants experiencing either Grade 3 or 4 cytokine release syndrome. •Proportion of participants with complete (CR) or partial response (PR) to CLIC-1901 •Meeting enrollment targets
NCT02790515	Provision of TCRγδ T Cells and Memory T Cells Plus Selected Use of Blinatumomab in Naïve T-cell Depleted Haploidentical Donor Hematopoietic Cell Transplantation for Hematologic Malignancies Relapsed or Refractory Despite Prior Transplantation	Recruiting	2	•Acute lymphoblastic leukemia (ALL) •Acute myeloid leukemia (AML) •Myeloid sarcoma •Chronic myeloid leukemia (CML) •Juvenile myelomonocytic leukemia (JMML) •Myelodysplastic syndrome (MDS) •Non-Hodgkin lymphoma (NHL)	•Drug: Anti-thymocyte globulin (rabbit) •Drug: Blinatumomab •Drug: Cyclophosphamide •Drug: Fludarabine •Drug: G-CSF •Drug: Melphalan •Drug: Mesna •Drug: Rituximab•Drug: Tacrolimus •Drug: Thiotepa •Biological: HPC, A Infusion •Device: CliniMACS •Drug: Sirolimus	•The number of patients engrafted by day +30 post-transplant •The number of patients experiencing blinatumomab permanent discontinuation due to toxicity •The estimate of cumulative incidence of relapse •The cumulative incidence of acute and chronic graft-versus-host disease (GVHD) •The cumulative incidence of transplant-related mortality
NCT04033302	Multi-CAR T Cell Therapy Targeting CD7-positive Malignancies	Recruiting	1/2	•T-cell acute Lymphoblastic leukemia •T-cell acute lymphoblastic lymphoma •Acute myeloid leukemia •NK cell lymphoma	•Biological: CD7-specific CAR gene-engineered T cells	•Safety of infusion •Clinical response
NCT04499339	A Phase I/IIa Clinical Trial to Assess Feasibility, Safety and Antitumor Activity of Autologous SLAMF7 CAR-T Cells in Multiple Myeloma	Recruiting	1/2	•Multiple myeloma	•Drug: SLAMF7 CAR-T	•Safety determination of the treatment with SLAMF7 CAR-T in phase I •Determination of the maximum tolerated dose (MTD) and the recommended phase IIa dose of SLAMF7 CAR-T in patients with MM •Safety determination of the treatment with SLAMF7 CAR-T in phases I and IIa •Evaluation of the efficacy, defined as overall response rate (ORR) after treatment with SLAMF7 CAR-T in patients with MM
NCT04109482	Trial to Evaluate the Safety and Efficacy of MB-102 in Patients with BPDCN.	Recruiting	1/2	•Blastic plasmacytoid dendritic cell neoplasm (BPDCN)	•Biological: MB-102 •Drug: Fludarabine •Drug: Cyclophosphamide	•Phase 1: Safety and tolerability as measured by the number of patients with treatment-related adverse events •Phase 1: Maximum tolerated dose (MTD) and recommended Phase 2 dose •Phase 2: Response Rate of patients with BPDCN •Phase 2: BPDCN—DOR •Phase 2: BPDCN—PFS •Phase 2: BPDCN—OS •Phase 2: BPDCN—MRD •Phase 2—Adverse events •Phase 2—Change from baseline in the European Organization for Research and Treatment (EORTC) QLQ-C 30 Version 3.0. •Phase 2—Change from baseline in the Functional Assessment of Cancer Therapy-Bone Marrow Transplant (FACT-BMT) Version 4.0. •Phase 2—Number of patients showing evidence of competent replication lentivirus
NCT03435796	Long-Term Follow-up Protocol for Subjects Treated With Gene-Modified T Cells	Recruiting	2/3	•Neoplasms	•Genetic: gene-modified (GM) T cell therapy	•Adverse events (AEs) •Tumor response status •Disease progression •Disease relapse •Overall survival •Health-related quality of life (HRQoL) •Height of pediatric subjects treated with GM T cells •Weight of pediatric subjects treated with GM T cells •Sexual maturation of pediatric subjects treated with GM T cells

**Table A2:** Select ClinicalTrials investigating safety of Vaccines—19 September 2021 [74].

NCT Number	Title	Status	Phase	Conditions	Interventions	Outcome
NCT01436968	Phase 3 Study of ProstAtak^®^ Immunotherapy With Standard Radiation Therapy for Localized Prostate Cancer Title Acronym: PrTK03	Recruiting	3	•Prostate cancer	•Biological: aglatimagene besadenovec + valacyclovir •Biological: placebo + valacyclovir	•Disease-free survival defined as the time from randomization until the date of the first failure event will be compared for the ProstAtak^®^ arm versus the placebo control arm. The analyses will be based on the intent to treat the population. •Prostate cancer-specific survival and overall survival will be compared for the ProstAtak^®^ arm versus the placebo control arm. •PSA nadir will be compared for the ProstAtak^®^ arm versus the placebo control arm. •Patient reported Health-Related Quality of Life outcomes would be collected using the Expanded Prostate Cancer Index Composite (EPIC-26) questionnaire. The change in QOL over time will be compared for the ProstAtak^®^ arm versus the placebo control arm. •The safety profile will be characterized by a collection of adverse event information and laboratory values during the treatment phase (until the completion of radiation). Data on late effects will be collected after radiation completion.
NCT03548571	Dendritic Cell Immunotherapy Against Cancer Stem Cells in Glioblastoma Patients Receiving Standard Therapy Title Acronym: DEN-STEM	Recruiting	2/3	•Glioblastoma	•Biological: dendritic cell immunization •Drug: adjuvant temozolomide	•Progression free survival •Overall survival •Patient reported quality of life, overall •Patient reported quality of life, brain-specific •Immunological response, skin •Immunological response, cellular •Adverse events
NCT04165317	Study of Sasanlimab (PF-06801591) in Combination With Bacillus Calmette-Guerin (BCG) in Participants With High-Risk Non-Muscle Invasive Bladder Cancer Title Acronym: CREST	Recruiting	3	•Non-muscle invasive •Bladder cancer	•Drug: PF-06801591 •Drug: Bacillus Calmette-Guerin	•Event free survival (Arm A compared to Arm C) •Event free survival (Arm B compared to Arm C) •Overall survival (Arm A compared to Arm C) •Overall survival (Arm B compared to Arm C) •Complete response in participants with CIS at randomization •Disease-specific survival •Health-related quality of life as measured by EORTC QLQ-C30 (European Organization for Treatment of Cancer Quality of Life Questionnaire for cancer patients) •Ctrough of PF-06801591 when combined with BCG (induction and maintenance or induction). Arms A and B only. •Incidence of ADA/Nab of PF-06801591 when in combination with BCG (induction and maintenance or induction). Arms A and B only. •Tumor sample biomarker status based on PD-L1 expression (high or low) •Duration of CR for participants with CIS at randomization •Time to recurrence of low-grade disease •Time to cystectomy •Health-related quality of life as measured by PTAB (Patient Treatment Administration Burden Questionnaire) •Percentage of participants with all causality and treatment-related treatment-emergent adverse events (TEAEs), serious adverse events (SAEs), and withdrawals due to TEAEs •Percentage of participants with laboratory abnormalities •Health-related quality of life as measured by EORTC QLQ-NMIBC24 (European Organization for Treatment of Cancer in patients with non-muscle invasion bladder cancer)
NCT03619252	Pneumococcal Vaccination of Multiple Myeloma Patients on Novel Agents	Enrolling by invitation	4	•Multiple myeloma •Pneumococcal infection •Febrile neutropenia •Pneumococcal pneumonia	•Biological: vaccination with pneumococcal conjugate vaccine (PCV13) •Drug: standard antibacterial prophylaxis	•Incidence of clinically/radiologically confirmed pneumonia and episodes of febrile neutropenia during one year period after initiation of novel agents. •Number of participants with treatment-related adverse events as assessed by CTCAE v4.0.
NCT00676507	Phase III Lucanix™ Vaccine Therapy in Advanced Non-small Cell Lung Cancer (NSCLC) Following Front-line Chemotherapy Title Acronym: STOP	Completed	3	•Lung neoplasm •Carcinoma non-small Cell lung cancer stage IIIA •Carcinoma non-small cell lung cancer Stage IIIB •Carcinoma non-small cell lung cancer Stage IV	•Biological: Lucanix™ •Other: placebo comparator	•Compare the overall survival of subjects with stage III or IV non-small cell lung cancer treated with belagenpumatucel-L (Lucanix™) vs placebo. •Evaluate the progression-free survival (PFS) of subjects treated with Lucanix™ compared to treatment within the Best Support Care control group. •Evaluate the quality of life (QOL) as determined by the Lung Cancer Symptom Scale (LCSS) compared to treatment within the Best Supportive Care control group. •Evaluate the time-to-progression of subjects treated with Lucanix™ compared to treatment within the Best Supportive Care control group. •Evaluate the best overall tumor response in subjects treated with Lucanix™ compared to treatment in the Best Supportive Care control group. •Evaluate the response duration in subjects treated with Lucanix™ compared to the Best Supportive Care control group. •Evaluate the rate of CNS metastases development in subjects treated with Lucanix™ as compared to the Best Supportive Care control group. •Adverse events of subjects treated with Lucanix™ will be compared to subjects in the Best Supportive Care control group.

**Table A3:** Select clinical trials investigating the safety of targeted therapy—19 September 2021 [74].

NCT Number	Title	Status	Phase	Conditions	Interventions	Outcome
NCT03673332	Elderly Cancer PatIents, Safety and qualiTy of Life Under immunOtheraPies Title Acronym: EPITOP-01	Recruiting	4	•Advanced or metastatic melanoma •Advanced or metastatic NSCLC	•Drug: immune-checkpoint inhibitors therapies	•Number of participants’ adverse events as assessed by CTCAE v5.0 •European Organization for Research and Treatment of Cancer Quality of Life Core Questionnaire (QLQ-C30) and Elderly Cancer Patients module (QLQ-ELD14), combined to compute a total score (between 44 and 128 points) •Geriatric data modifications under treatment •Compare patients and clinician symptoms reporting •Progression-free survival •Overall survival •Correlation between toxicity and efficacy •Rate of grade 3 to 5 adverse events 18 weeks after treatment initiation
NCT02344472	Detect V/CHEVENDO (Chemo vs. Endo)	Recruiting	3	•Metastatic breast cancer	•Drug: Pertuzumab •Drug: Trastuzumab •Drug: Capecitabine •Drug: Paclitaxel •Drug: Vinorelbine •Drug: Docetaxel •Drug: Exemestane •Drug: Letrozole •Drug: Anastrozole •Drug: Fulvestrant •Drug: Ribociclib •Drug: Nab-Paclitaxel •Drug: Eribulin •Drug: Leuprorelin •Drug: Goserelin	•Number of participants with adverse events •Quality-adjusted survival •Overall response rate (ORR) •Incidence of central nervous system (CNS) metastases and their control rate •Analysis of quality of life, presence and number of circulating tumor cells (CTC) at different time points •Evaluation of all reported events and all grades in both treatment arms (chemotherapy and endocrine therapy) •Disease control rate (DCR) •Progression-free survival (PFS) •Overall survival (OS)
NCT02889692	Clinical Study of Chinese Medicine Plus Targeted Therapy Maintenance in Advanced Pulmonary Adenocarcinoma	Completed	3	•Cancer	•Drug: Yi Qi Fang •Drug: Yang Yin Fang •Drug: Yi Qi Yang Yin Fang •Drug: placebo granules •Drug: Erlotinib^®^ •Drug: Gefitinib^®^ •Drug: Icotinib^®^	•Overall survival (OS) •Progression-free survival (PFS) •Objective response rate (ORR) •Quality of life (QOL)
NCT03949231	Infusion of PD1/PDL1 Inhibitor Via Neck Artery Versus Vein for Immunotherapy of Head/Neck Cancers (HNC)	Recruiting	3	•Head/neck neoplasm	•Drug: PD1/PDL1 inhibitor	•Overall survival •Progression-free survival •Adverse event rate
NCT04564157	NADIM-ADJUVANT: New Adjuvant Trial of Chemotherapy vs Chemo-immunotherapy	Recruiting	3	• Non-small cell lung cancer •Adjuvant chemotherapy	•Drug: Carboplatin •Drug: Paclitaxel •Drug: Nivolumab	•The disease-free survival •Overall survival •Incidence of treatment-emergent adverse events (safety and tolerability)
NCT00861614	Study of Immunotherapy to Treat Advanced Prostate Cancer	Completed	3	•Prostate cancer	•Drug: Ipilimumab •Drug: placebo	•Overall survival (OS) •Overall survival rate •Progression free survival (PFS) •Pain response •Duration of pain response •Number of participants with severe adverse events (AEs), serious adverse events (SAEs), treatment-related AEs, deaths, discontinuation of study drug due to AEs, immune-related adverse events (irAE), and immune-mediated adverse reaction (imAR) •Time to onset of Grade 3 or 4 immune-related adverse event (irAE) •Time to resolution of Grade 3 or 4 immune-related adverse event (irAE) •Time to onset of Grade 3 to 5 immune-mediated Adverse reaction (imAR) •Time to resolution of Grade 3 to 5 to Grade 0 immune-mediated adverse reactions (imARs) to Grade 0 •Number of participants with worst on-study hematology common toxicity criteria (CTC) grade and shift From baseline •Number of participants with worst on-study liver common toxicity criteria (CTC) grade and shift from baseline •Number of participants with worst on-study serum chemistry common toxicity criteria (CTC) Grade and shift From baseline •Number of participants with worst on-study renal function common toxicity criteria (CTC) grade and shift from baseline
NCT01057810	Phase 3 Study of Immunotherapy to Treat Advanced Prostate Cancer	Completed	3	•Prostate cancer	•Drug: Ipilimumab •Drug: placebo	•Overall survival (OS) •Time progression-free survival (PFS) time •Time to subsequent non-hormonal cytotoxic therapy •Time to pain progression •Number of participants who died or had adverse events (AEs), serious adverse events (SAEs), immune-related AEs (irAEs), or immune-mediated adverse reactions (imARs) •Number of treated participants with Grade 3 or 4 clinical laboratory abnormalities
NCT04821778	Chemoradiotherapy in Esophageal or Esophagogastric Junction Cancer	Recruiting	3	•Esophagus cancer •Esophagogastric junction cancer •Chemoradiation •Targeted therapy •Immunotherapy •Chemotherapy effect	•Radiation: radiotherapy •Drug: platinum-based chemotherapy •Drug: Paclitaxel based chemotherapy •Drug: immunotherapy •Drug: 5-FU analog-based chemotherapy •Drug: Nimotuzumab	•Overall survival •Progression free survival •Number of participants with acute and late toxicities of radiotherapy, chemotherapy, and immunotherapy •Pathological response rate •R0 resection rate •Locoregional recurrence-free ser •Distant metastasis-free survival
NCT04821843	Neoadjuvant Treatment Modalities in Esophageal Cancer	Recruiting	3	•Esophageal cancer •Chemotherapy effect •Chemoradiation •Surgery •Targeted therapy •Immunotherapy •Esophagogastric junction cancer	•Drug: Platinum-based chemotherapy •Drug: Paclitaxel based chemotherapy •Radiation: radiotherapy •Procedure: surgery •Drug: immunotherapy •Drug: 5-FU analog-based chemotherapy •Drug: Nimotuzumab	•Overall survival •Progression-free survival •Number of participants with acute and late toxicities of radiotherapy, chemotherapy and immunotherapy •Pathological response rate •R0 resection rate •Locoregional recurrence-free survival •Distant metastasis-free survival
NCT03793179	Testing the Timing of Pembrolizumab Alone or With Chemotherapy as First-Line Treatment and Maintenance in Non-small Cell Lung Cancer	Recruiting	3	•Non-squamous non-small cell carcinoma •Stage IIIB lung cancer AJCC v8 •Stage IIIC lung cancer AJCC v8 •Stage IV lung cancer AJCC v8 •Stage IVA lung cancer AJCC v8 •Stage IVB lung cancer AJCC v8	•Drug: Carboplatin •Biological: Pembrolizumab •Drug: Pemetrexed	•Overall survival (OS) •Progression-free survival (PFS) •Best objective response •Incidence of adverse events •PD-L1 positivity
